# Unmet needs in pneumonia research: a comprehensive approach by the CAPNETZ study group

**DOI:** 10.1186/s12931-022-02117-3

**Published:** 2022-09-10

**Authors:** Mathias W. Pletz, Andreas Vestergaard Jensen, Christina Bahrs, Claudia Davenport, Jan Rupp, Martin Witzenrath, Grit Barten-Neiner, Martin Kolditz, Sabine Dettmer, James D. Chalmers, Daiana Stolz, Norbert Suttorp, Stefano Aliberti, Wolfgang M. Kuebler, Gernot Rohde

**Affiliations:** 1 Institute of Infectious Diseases and Infection Control, Jena University Hospital - Friedrich Schiller University Jena, Jena, Germany; 2 CAPNETZ STIFTUNG, Hannover, Germany; 3 Department of Pulmonary and Infectious Diseases, Nordsjællands Hospital, Hillerød, Denmark; 4 Division of Infectious Diseases and Tropical Medicine, Department of Medicine I, Medical University of Vienna, Vienna, Austria; 5 Department of Infectious Diseases and Microbiology, University Hospital Schleswig-Holstein, German Center for Infection Research (DZIF), Partner Site Hamburg-Lübeck-Borstel-Riems, Lübeck, Germany; 6 Department of Infectious Diseases and Respiratory Medicine, Charité - Universitätsmedizin Berlin, Berlin, Germany; 7 Biomedical Research in Endstage and Obstructive Lung Disease Hannover (BREATH), German Center for Lung Research (DZL), Hannover, Germany; 8 Division of Pulmonology, Medical Department I, University Hospital Carl Gustav Carus, Dresden, Germany; 9 Department of Diagnostic and Interventional Radiology, Hannover Medical School, Hannover, Germany; 10 Scottish Centre for Respiratory Research, Ninewells Hospital and Medical School, University of Dundee, Dundee, DD1 9SY UK; 11 Clinic of Respiratory Medicine and Pulmonary Cell Research, University Hospital, Basel, Switzerland; 12 Department of Pneumology, University Medical Center, Freiburg, Germany; 13 German Center for Lung Research (DZL), Berlin, Germany; 14 Department of Biomedical Sciences, Humanitas University, Via Rita Levi Montalcini 4, 20072 Pieve Emanuele, Milan, Italy; 15 IRCCS Humanitas Research Hospital, Respiratory Unit, Via Manzoni 56, 20089 Rozzano, Milan, Italy; 16 Institute of Physiology, Charité – Universitätsmedizin Berlin, Berlin, Germany; 17 Department of Respiratory Medicine, Medical Clinic I, University hospital, Goethe University Frankfurt, Theodor-Stern-Kai 7, 60596 Frankfurt/Main, Germany

**Keywords:** CAP pathogens, Next generation sequencing, Imaging, Biomarkers, Pneumococcal vaccines, Universal influenza vaccine, Translational research, Personalized medicine, Immune-compromised host, Machine learning, The “omics” approach

## Abstract

**Introduction:**

Despite improvements in medical science and public health, mortality of community-acquired pneumonia (CAP) has barely changed throughout the last 15 years. The current SARS-CoV-2 pandemic has once again highlighted the central importance of acute respiratory infections to human health. The “network of excellence on Community Acquired Pneumonia” (CAPNETZ) hosts the most comprehensive CAP database worldwide including more than 12,000 patients. CAPNETZ connects physicians, microbiologists, virologists, epidemiologists, and computer scientists throughout Europe. Our aim was to summarize the current situation in CAP research and identify the most pressing unmet needs in CAP research.

**Methods:**

To identify areas of future CAP research, CAPNETZ followed a multiple-step procedure. First, research members of CAPNETZ were individually asked to identify unmet needs. Second, the top 100 experts in the field of CAP research were asked for their insights about the unmet needs in CAP (Delphi approach). Third, internal and external experts discussed unmet needs in CAP at a scientific retreat.

**Results:**

Eleven topics for future CAP research were identified: detection of causative pathogens, next generation sequencing for antimicrobial treatment guidance, imaging diagnostics, biomarkers, risk stratification, antiviral and antibiotic treatment, adjunctive therapy, vaccines and prevention, systemic and local immune response, comorbidities, and long-term cardio-vascular complications.

**Conclusion:**

Pneumonia is a complex disease where the interplay between pathogens, immune system and comorbidities not only impose an immediate risk of mortality but also affect the patients’ risk of developing comorbidities as well as mortality for up to a decade after pneumonia has resolved. Our review of unmet needs in CAP research has shown that there are still major shortcomings in our knowledge of CAP.

## Introduction

In 1918, Sir William Osler observed that pneumonia had replaced tuberculosis as the leading cause of death in Europe and described pneumonia as the ‘‘Captain of the men of death’’ [1].

Even one century later, pneumonia remains a major health concern and lower respiratory tract infections continue to be the leading infectious cause of death with more than 2.3 million deaths and more than 91 million years of life lost in 2016 [2]. With the emergence of the SARS-CoV-2 pandemic the central importance of acute respiratory infections to human health has once again been highlighted.

Pneumonia is defined as an acute infection of the pulmonary parenchyma and it is commonly classified by the “pneumonia triad” into community-acquired pneumonia (CAP) as the most common form of acquisition, hospital-acquired pneumonia (HAP) including ventilator-associated pneumonia (VAP) and pneumonia in the immunocompromised host [3, 4]. The clinical presentation and severity of pneumonia is diverse and usually categorized as mild, moderate or severe in major guidelines [5–7].

The annual incidence of CAP requiring hospitalization ranges from 1.1 to 8.9 per 1000 inhabitants [8–11] and the 30-day readmission rate ranges from 12 to 25% [12–15]. In patients with CAP who do not need hospital admission the mortality rate is ≤ 1% [16–18] while in hospitalized patients CAP the short-term mortality ranges from 8 to 14% [8, 10, 11, 19–23]. Further, several studies have shown that long-term mortality is excessive after CAP [24–29]. This underlines that CAP imposes a significant impact on the healthcare system and because of the aging population and the associated increase in comorbidities predisposing for CAP, it is likely that CAP will continue to be a major health issue in the years to come.

In 1999, the German Ministry of Education and Research published a call to initiate an excellence competition for competence centres in infectious diseases. Through this call the “network of excellence on Community Acquired Pneumonia” (CAPNETZ) was founded in 2001 [30]. CAPNETZ connects physicians, microbiologists, virologists, epidemiologists and computer scientists within clinical centres in Germany, Switzerland, Austria, The Netherlands, Denmark and Italy [31]. Today CAPNETZ offers the most comprehensive CAP database worldwide with an associated biobank for samples and pathogens including more than 12,000 patients [31]. Currently, the CAPNETZ database contains information on adult patients and consequently the pneumonia research within CAPNETZ has been focusing on adults. Pneumonia in children is also a major issue but an entity of its mown due to different pathogens, less burden of comorbidities etc. Recognizing the significance of pneumonia in children CAPNETZ has expanded their collaboration and begun inclusion of children into the pedCAPNETZ cohort [32]. The pedCAPNETZ cohort is still under construction and therefore the focus of this study is on adults only.

To discuss and identify which future research areas should have the highest priority in adult CAP research, CAPNETZ invited both internal and external CAP experts to participate in a 2-day scientific retreat. This paper summarizes the main research topics discussed, and highlights areas of future research, which deserve high priority to improve the understanding of CAP in order to develop novel prophylactic and therapeutic measures and ultimately reduce the burden of CAP.

## Procedures

**Fig. 1:**
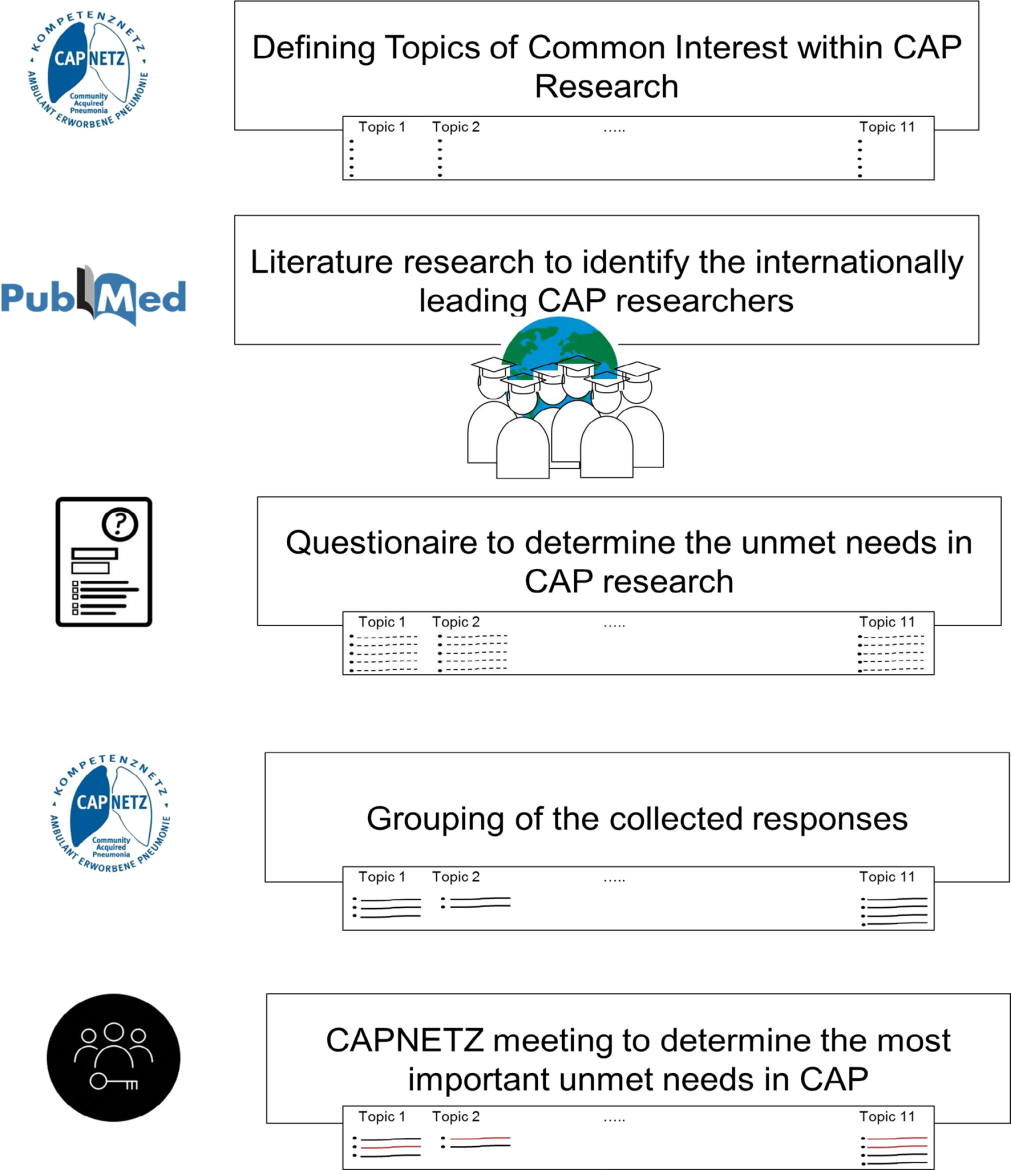
Flowchart for the identification of the unmet needs in community-acquired pneumonia research

The identification of unmet needs in CAP research followed a four-step procedure as depicted in Fig. 1. In brief, research members of CAPNETZ were asked to identify important issues to be addressed within basic, clinical and translational research. Then the top 100 experts in the field of CAP research were asked for their insights about the unmet needs in CAP research (Delphi approach). The top 100 experts were determined by CAPNETZ as the researchers with the most publications in the web of knowledge database over the past 5 years marked with “community-acquired pneumonia”. Only original publications, reviews and editorials were taken into consideration. Thirty-three of the 100 experts contributed with their views on the unmet needs in CAP research. Finally, the identified unmet needs in CAP research were discussed at a 2-day scientific retreat.

Since the SARS-CoV-2 pandemic emerged after the scientific retreat was held no unmet needs where identified prior to the meeting. However, due to the significant impact of the SARS-CoV-2 pandemic, sections on the Covid-19 disease have been added post hoc.

## Results

### Identified topics covering unmet needs in CAP research

#### Detection of causative pathogens

##### Current setting

Defining the causative pathogen in patients with pneumonia is relevant for ensuring the best-suited antimicrobial treatment. However, microbial diagnosis of pneumonia is still based on culture in most hospitals worldwide and a causative pathogen is only detected in the minority of patients. In a recent CAPNETZ study, bacteraemia rates in CAP were 0.5% in outpatients, 7.8% in hospitalized patients, 12.4% in patients admitted to an intensive care unit (ICU), and a relevant proportion of patients with bacteraemia and CAP (34.6%) was afebrile [33]. With the rapid emergence of novel diagnostic techniques (e.g. isothermic multiplex-PCRs for point of care testing, microbiome analysis, host transcriptomics and metagenomics), the question arises if these techniques will provide an added value to the currently available and established diagnostic methods and whether they will change management.

##### Unmet needs

Define an improved gold standard for microbial diagnosis of community-acquired pneumonia: Currently, microscopy and culture of lower respiratory tract specimens as well as blood cultures, detection of urine antigen detection and serology are routinely used to complement thorax imaging results in diagnosing pneumonia. However, most tests (particularly serology) lack sensitivity and specificity, while tests with a high sensitivity, such as PCR for non-influenza respiratory viruses may currently not properly translate into clinical relevance [34, 35]. No internationally accepted standard for microbiological tests in CAP has been defined, both in terms of including novel PCR-based techniques but also regarding the quality of the required respiratory samples [36]. There is a need for a consented diagnostic standard that also serves as comparison for new diagnostic techniques [37].

#### Next generation sequencing for guidance of antimicrobial treatment

##### Current setting

With the emergence of resistant or multidrug resistant (MDR) pathogens and increasing knowledge about potentially harmful or protective microbiomes [38], antibiotic management is becoming more complex [39]. Antibiotic stewardship is, therefore, a key priority [40] fostering pathogen directed treatment and avoiding excessive empiric broad spectrum antimicrobial therapy that increases the risk of complications, antibiotic resistance and mortality.

Metagenomics offers a potential solution. Most studies utilizing “microbiome” or metagenomics techniques to date have used target gene approaches such as sequencing amplicons of the 16s rRNA subunit [41]. As a more rapid technique, nanopore sequencing allows a much more in-depth data acquisition and analysis of viral and bacterial pathogens in real time [42]. The INHALE study from the UK showed that nanopore sequencing could provide results within 6 h of sampling from sputum with 96.6% sensitivity compared to culture [42]. This method also presented data on antibiotic resistance genes. Currently, methods to comprehensively describe bacterial, fungal and viral pathogens as well as the underlying microbiome in the same sample are still limited but technology is advancing rapidly.

##### Unmet needs

Metagenomic research has, to date, been conducted in relatively small studies in limited geographical locations [42]. Much larger studies are required to fully understand the limitations and added value of metagenomic approaches.

Studies show that molecular techniques out-perform culture in identification of bacterial pathogens [38, 42], but studies to demonstrate that implementation of these techniques improves clinical practice are lacking. There are remarkably few studies in which antibiotic choice, antibiotic duration or antibiotic spectrum are reduced or improved by the implementation of such techniques. Randomized controlled trials should be conducted to demonstrate the value of new techniques to improve CAP management.

Clinicians are not bioinformatics-scientists. Therefore, automated informatics tools that can rapidly analyse sequencing data and provide results to clinicians in a usable format are required. Antibiotic resistance genes may be detected by sequencing but their relationship to phenotypic resistance in bacteria is not always known, particularly in Gram-negatives.

#### Imaging diagnostics

##### Current setting

The primary role of imaging in CAP is to confirm the clinical diagnosis. The major diagnostic challenges are the correct identification of opacities and consolidations as being evoked by infectious *versus* non-infectious differential diagnoses. Different methods are available with chest radiography being the most abundantly used [43]. However, its low sensitivity and specificity leads to a high false negative and false positive rate, respectively, depending on the infiltrate’s position [44, 45]. Computer tomography (CT), therefore, is a useful addition to conventional radiography because of three-dimensional imaging with enhanced spatial resolution [44–48]. Due to higher radiation exposure and costs, however, CT is currently only used in selected cases [43, 49]. Further, lung ultrasonography may be an adjuvant resource to accurately diagnose CAP [50].

##### Unmet needs

Pathogen prediction through imaging analysis: Although radiographic findings do currently not allow the diagnosis of a specific pathogen, a differential diagnosis may be possible using radiological pattern recognition. Deep learning approaches may help to identify patterns that are indicative of specific viral or bacterial CAP. Integrating clinical data available at time of diagnosis may further improve such an approach.

Pre-test probability for general practitioners for chest radiography: Although chest radiographs are routinely used, the efficacy as a diagnostic tool has not been determined. The reduction of unnecessary chest radiographs particularly in the outpatient setting would decrease healthcare costs, and radiation exposure. However, even in the “Prospective Cough Complication Cohort Study (3C)”, where patients with acute cough received chest radiographs, the positive predictive value of at least one of four clinical signs of CAP (temperature > 37.8 °C, crackles on auscultation, oxygen saturation < 95%, heart rate > 100/min) for a radiography-confirmed pneumonia was low at around 20% [51]. In another European multicentre study including more than 3000 patients with acute cough, the addition of C-reactive protein > 30 mg/l to clinical signs of pneumonia (absence of runny nose and presence of breathlessness, crackles and diminished breath sounds on auscultation, tachycardia, and fever) improved the diagnostic classification [52]. However, further enhancing the pre-test probability for CAP would be necessary to provide a more reliable guide to general practitioners to identify patients with respiratory tract infections other than CAP where radiographs are not necessary.

#### Biomarkers

##### Current setting

Biomarkers may be an effective way to identify patients with severe infection and to monitor and eventually predict the course of infection. In CAP, biomarkers may be indicators of inflammation (local or systemic), may be released specifically after lung injury due to infection or originate from the genome of either the host or the pathogens. Numerous biomarkers have been investigated including pro-atrial natriuretic peptide [53], pro-vasopressin [53], cortisol [54], glucose [55], glycaemic gap [56], neutrophil extracellular traps [57], proadrenomedullin [58], Angiopoietin [59] etc. [60, 61] but C-reactive protein and procalcitonin remain the most widely used biomarkers [60] even though they both have well known shortcomings [60]. Additionally, cardiac troponins, biomarkers for cardiac injury, may identify patients with cardiac complications of CAP that need special attention during follow-up [62, 63]. Since coronary artery calcium (CAC), a marker of coronary atherosclerotic burden, is higher in patients already at increased atherosclerotic cardiovascular risk after pneumonia as compared to similar patients without a pneumonia event, assessment of CAC by CT may also be of predictive value [64].

##### Unmet needs

As the research on immune-modulatory therapies intensifies, so will the search for the appropriate biomarkers for severity and treatment response [65]. However, the heterogeneity among patients with CAP in terms of onset of symptoms, clinical presentation, severity of disease, causative pathogen(s), comorbidities, genetic disposition etc. is vast. The discovery of a single common biomarker for CAP is therefore unlikely. Instead, combining the different sources of “biomarkers” into a systems-medicine approach reflects the complexity of CAP and could provide new insights, accurate clinical diagnosis, prediction of the severity of disease and help targeting specific adjuvant treatments. The recent advances in the field of metabolomics, genomics, epigenomics, transcriptomics, proteomics and microbiomics offer respective opportunities [60, 66].

#### Risk stratification

##### Current setting

Identifying low risk-patients that can be treated in an outpatient setting enhances patient satisfaction and reduces costs. CRB-65 is frequently used as a first step but should be supplemented by oxygenation status, assessment of instable comorbidities and functional parameters [67]. Identifying high-risk patients allows early application of intensified management, prevents organ failure and, thereby, improves prognosis. ATS/IDSA minor criteria supplemented by lactate are recommended tools [68, 69]. Recommendations for early high-risk stratification are available but have not yet been fully implemented. Although there is a five percent risk of death within the first post-discharge weeks [70], there is only limited data available on risk stratification to predict post-discharge complications.

##### Unmet needs

Harmonization with sepsis risk stratification and implementation of prediction tools: Sepsis is a major complication in CAP. Current sepsis recommendations employ a risk stratification strategy with similarities to CAP risk stratification by using a simple screening score (quickSOFA, qSOFA) and a more specific high-risk prediction score (SOFA) [71]. However, the scores and respective breakpoints (e.g. respiratory rate in qSOFA vs. CRB-65) are different from those recommended for CAP [72]. Moreover, the qSOFA with a cut off of ≥ 2 misses more than half of CAP patients whose condition will deteriorate during the course of the disease [73]. For optimal implementation of risk stratification concepts in busy emergency departments a harmonization of both concepts is needed, and prospective (cluster-randomized) clinical studies to implement recommendations with clinical outcome endpoints are necessary.

Individualization of management-based risk stratification: Today, medicine is heading towards the broad application of state-of-the-art medical technologies including “omics” approaches, which might allow more tailored treatment approaches based on a better understanding of a patient’s individual state of health [74]. In addition, machine learning could provide algorithms that predict the patient’s individual risk to deteriorate and will allow monitoring treatment responses based on routinely measured markers [75].

Risk factors for and management strategies against early post-discharge complications: Studies show high re-admission or even early death rate after discharge from hospital because of CAP [12, 76]. So far, only little information is available on risk factors for post-discharge events, including infection- and non-infection-related complications, re-hospitalization or death. There is the need to determine the different affected patient groups and to define target groups that require interventions.

Stratification strategy for the coverage of multidrug resistant pathogens: In Europe, MDR pathogens are rare in CAP, but still can complicate the choice of empiric antibiotic therapies and may result in poor outcomes [77]. However, optimal individual MDR risk prediction is not known as existing scores lack accuracy and external validation.

#### Antiviral and antibiotic treatment

##### Current setting

The early initiation of antibiotic therapy has been shown to provide a survival benefit [78]. Treatment is typically started empirically, prior to the identification of the causative pathogen. Bacteria are considered to be the primary causative pathogens in CAP and antibiotics are the mainstay of CAP treatment.

Treatment guidelines differ for outpatients and only little information is available on CAP-outpatients. In the hospital, either antibiotic monotherapy or combination therapy is applied but up to now no significant differences in outcome apart from adverse effects have been observed [79]. Most antibiotics are administered systemically, either through intravenous or oral application. The role of aerosolized antibiotics has so far been only investigated for hospital-acquired (HAP) and ventilator-associated pneumonia, but not for CAP [80].

Viral pathogens are increasingly recognized as a cause of pneumonia, especially among immunocompromised patients. Although more than 20 viruses have been linked with CAP, antiviral drugs were only available for the treatment of influenza or respiratory syncytial virus (RSV) pneumonia [81] before COVID-19. Since the occurrence of SARS-CoV-2 existing antiviral drugs such as Remdesivir and lopinavir–ritonavir have been evaluated for the treatment of Covid-19 [82]. However, lopinavir–ritonavir has not shown any beneficial effect in treating Covid-19 and the effect of Remdesivir in treating Covid-19 is still questionable [82]. The specifically developed antiviral Paxlovid may hold promise but peer-reviewed data are currently lacking [83].

Secondary bacterial pneumonia is frequently observed in viral pneumonia [84, 85] and influenza patients often receive preventive antibiotic treatment [86]. However, the pathophysiologic mechanisms promoting a co-infection with bacterial and viral pathogens are not fully understood. Notably, the frequency of bacterial co-infections in Covid-19 patients appears to be low [87].

##### Unmet needs

Monitoring of ß-lactam antibiotic and macrolide antibiotic treatment and development of resistance: ß-lactams have been the “antibiotic backbone” of antimicrobial therapy of pneumonia for decades [88]. However, increasing resistance rates are beginning to limit the utility of this antibiotic class. Drug-resistant Gram-negative pathogens associated with pneumonia have been identified with increasing frequency and a variety of resistance patterns. Among the “atypical” agents there is also several reports of increasing macrolide resistance in *M. pneumoniae* infections throughout the world [89–91]. Antibiotic resistances show a broad variety between regions. Treatment guidelines, therefore, need to be updated and validated based on local epidemiological data [92].

Development of new antibiotics vs. improved usage of available antibiotics: Although the number of newly approved antibiotics has tripled in the past 6 years after a 90% decrease between 1983 and 2012, MDR bacteria pose an increasing threat [93]. Mis- and overuse of empirically prescribed broad-spectrum antibiotics have led to a significant increase in resistance although a great diversity has been observed in different countries [94]. The timely differentiation between bacterial and viral pneumonia may support the physician in avoiding medically not indicated antibiotic administration. In CAP, development of new antibiotics and improving diagnostic-guided therapy is warranted.

Overview of the treatment of CAP in an outpatient setting: A high proportion of CAP patients is treated in an outpatient setting [52, 70]. A comprehensive overview of existing real-life principles that guide treatment in an outpatient setting will allow the formulation of improved guidelines taking the medical environment and available methods into account.

#### Adjunctive therapy

##### Current setting

An excessive inflammatory response seems to be partly responsible for treatment failure in some patients and has been associated with poor clinical outcomes [95]. Different immunomodulatory and barrier-enhancing agents have been discussed and tested for potential adjunctive therapy to antimicrobial agents in the treatment of CAP. Classical approaches have focused on corticosteroids and immunoglobulins [95], while the effects of adrenomedullin and angiopoietin-1 have been investigated in more experimental approaches [59, 96]. Especially corticosteroids have been the subject of potential adjuncts to conventional CAP treatment [95]. They are the most used anti-inflammatory drugs and modulate a wide range of physiological processes, but their efficacy has been discussed controversially in CAP treatment [97]. In hospitalized patients with Covid-19 and who requires oxygen, corticosteroid treatment improves survival [98] and is therefore recommended [82].

Further, due to the hyperinflammation state seen in some patients with Covid-19 several immunomodulatory agents, with the intend to block the inflammatory pathway, have been evaluated [82, 99]. Fare from all has proven effective but in hospitalized patients with Covid-19 and in need of oxygen treatment, IL-6 inhibitors may reduce the risk of mechanical ventilation or death [82]. Likewise treatment with Janus kinase (JAK) inhibitors may reduce the risk of respiratory failure and death [100, 101]. Inhibition of the IL-1 pathway may also be associated with reduced mortality in patients with Covid-19 [102] although conflicting results exists [103, 104].

As the understanding of the underlying pathophysiology improves, specifically tailored agents for immunomodulatory therapy will likely help to avoid adverse outcomes for specific patient groups.

##### Unmet needs

Identification of patients that benefit most from macrolides: In addition to antimicrobial effects, macrolides also have an immunomodulatory effect [105, 106]. Several studies have suggested a benefit of adding macrolides to a β-lactam in the empirical treatment, although the existing literature is conflicting [105, 107–109]. It is largely unclear, which patient groups benefit most from an adjunctive macrolide therapy since the effects of macrolides appear to be influenced by the presence of bacteria [106, 110] and by the susceptibility of the host to develop cardiac side effects associated with macrolide treatment [111].

A recent CAPNETZ study has used a machine learning approach to identify patients who benefit most from macrolide treatment [112]. Such a personalized approach is important for improved management and disease outcome. However, results need to be confirmed in a randomized controlled trial.

Immunoglobulins: Immunoglobulins have been proposed as a promising adjunctive therapy option for severe sepsis, but have only been investigated in small studies [113]. Both, IgG and IgM levels have been shown to be higher during convalescence in pneumonia [114]. Additionally, patients with severe CAP admitted to ICU showed lower levels of immunoglobulins than non-ICU patients [115]. Since therapeutic formulations of immunoglobulins are available further insights into the changes of serum levels of immunoglobulins and IgG subclasses during the course of the disease are of scientific and therapeutic interest.

Pathogen-directed strategies: In addition to antibiotic treatment, a pathogen-directed strategy may include blockers of pathogenicity, virulence, or toxins. These may be small molecule inhibitors or monoclonal antibodies [116]. Additional use of adoptive cell therapy might be possible as well as the use of phages or phage products. In the ongoing pandemic of Covid-19 treatment with monoclonal antibodies against the SARS-CoV-2 spike protein may reduce the risk of Covid-19-related hospitalization and death in ambulatory patients [117] and in hospitalized patients with Covid-19 and who are seronegative, treatment with monoclonal antibodies may reduce mortality [118].

Host-directed strategies: Alternative strategies comprise of specifically stimulating early local immune responses against pathogens, dampening particular components of the local and systemic immune responses to avoid tissue injury, increasing tissue resilience and improving resolution of inflammation and tissue repair.

#### Vaccines and prevention

##### Current setting

Vaccines against pneumococci and influenza virus, the most frequent bacterial and viral causes of CAP, are available. Influenza vaccination has been shown to reduce the number of severe CAP cases and improved overall long-term survival in patients with CAP during influenza seasons [119]. The known mechanistic link between cardiovascular events and pneumonia may be the cause for the reported cardioprotective effect of vaccines against influenza and pneumococci [120–122].

Recently, a quadrivalent influenza vaccine that includes both influenza B lines, i.e. Yamagata and Victoria, has been made available for clinical use [123]. It has already been recommended as the primary influenza vaccine instead of the trivalent influenza vaccines in several countries. The investigational universal influenza vaccine candidate, FLU-v, has entered phase 3 clinical trials [124] after demonstrating immunogenicity and safety in a recent phase 2b study [125].

Two types of pneumococcal vaccines are used: pneumococcal conjugate vaccines (PCVs) and pneumococcal polysaccharide vaccines (PPVs). Although PPV has been shown to prevent pneumococcal bacteraemia, its protective effect against non-invasive pneumococcal pneumonia and its effectiveness in the immune-compromised host are limited [126–128]. Infant vaccination programs with PCV have substantially decreased the contained serotypes by herd protection effects [129]. However, the decrease of vaccine serotypes was compensated by non-vaccine serotypes (replacement effect), that comprised sometimes even the same pneumococcal clone, which has switched its capsule to another serotype to evade the selective pressure of the vaccine [130]. Furthermore, strong herd protection effects in invasive and non-invasive pneumococcal CAP seen after the global implementation of the 7-valent conjugate vaccine (PCV7) [129, 131] were not reproduced after substitution of PCV7 by the 13-valent conjugate vaccine (PCV13) [132, 133]. Particularly, serotype 3, which was included into PCV13, seems not to be affected by herd protection effects. Serotype 3 has been associated with disease severity, i.e. higher rate of patients with hospital admission and oxygen support, and has become one of the leading serotypes in adult CAP [133, 134]. The decreased efficacy against serotype 3 as well as the lack of herd protection of this major serotype is not completely understood. One plausible hypothesis includes capsular shedding after binding of antibodies [135].

##### Unmet needs

Optimal use for pneumococcal polysaccharide vaccines and pneumococcal conjugate vaccines: Although the two vaccines PPV23 and PCV13 share some serotypes they generate different immune responses [136]. While both vaccines generate antibodies against pneumococcal capsular antigens, only PCV13 induces a T-cell-dependent response. The broad landscape of different pneumococcal vaccination recommendation worldwide (i.e. PPV or PCV or “sequential vaccination” with PCV followed by PPV) reflects uncertainty on the optimal use of these vaccines. Furthermore, simultaneous application of both pneumococcal vaccines (PCV and PPV) is currently investigated for the first time in an ongoing randomized controlled trial to improve immunological response (pneumococcal serotype specific B-cells and humoral immune response) in the elderly [137]. More research is needed on the topic to provide vaccination recommendations especially for patients with certain underlying medical conditions such as respiratory, hepatic or renal comorbidities and immunosuppression as well as the optimal time for re-vaccination [138].

Novel vaccines: Like a universal influenza vaccine, a serotype independent pneumococcal vaccine would decrease the global CAP burden tremendously. Currently, a 15-valent PCV was announced in the US and a 20-valent PCV is close to market license [139]. However, experience with PCV7 and PCV13 have shown that pneumococcal evolution is highly dynamic and that non-vaccine serotypes will emerge and fill the niche created by PCV-induced reduction of vaccine serotypes. Since the inclusion of even more serotypes into a novel vaccine is limited, a serotype independent vaccine targeting e.g. surface proteins would represent a major breakthrough [140]. Also, a more effective—even singular—vaccine against serotype 3 would decrease the burden of CAP tremendously.

The progress in microbiological diagnostics—and also the recent SARS-CoV-2 pandemics- has uncovered the burden of non-influenza respiratory viruses in CAP. Particularly, RSV seems to expose a substantial burden on adult CAP. Therefore, a RSV vaccine is highly desirable but remains a challenge [141], since earlier RSV vaccines had no efficacy and even seemed to aggravate disease [142].

#### Systemic and local immune response

##### Current setting

In order to facilitate a rapid and efficient immune response against invading microbial pathogens, the lung combines different defence strategies including anatomic, mechanical, humoral, and cellular mechanisms aiming towards the rapid expulsion of pathogens [143]. The lung is a fragile organ that is finely designed for gas exchange, so that an excessive immune response may itself be damaging and lead to irreparable tissue damage that might be lethal [144, 145]. Many anti-inflammatory strategies have failed to improve survival in pneumonia [146]. It seems that more specific anti-inflammatory strategies, subphenotyping of patients, and precise timing are crucial to achieve beneficial modulation of the inflammatory response. Differences in immune responses may result from genetic predispositions that influence immunomodulation [147, 148]. Deciphering the mechanisms of inflammatory response in respiratory infection would allow the identification of different inflammatory phenotypes.

##### Unmet needs

Determination of different inflammatory phenotypes for personalized medicine: Despite sharing the same underlying pathogen, in some patients CAP manifests as a serious disease while in others the course of disease is mild. The susceptibility to infection as well as CAP severity is most likely a phenotypical trait determined by uncountable pathogen- and host-specific factors, including polymorphisms in many collaborating genes in otherwise healthy persons [149], immunosenescence, pregnancy, lung diseases, immunodeficiency, and specific therapies for preceding diseases or the CAP itself, to name a few. Therefore, specific conditions need specific strategies for promising personalized adjunctive immunomodulatory therapy.

Pulmonary long-term consequences of CAP: While most patients return to normal lung function following pneumonia within weeks to months, some fail to recover ad integrum due to pleura-involvement or parenchymal alteration, and some may be at an increased risk of developing chronic non-infectious lung inflammation including cryptogenic organizing pneumonia (COP) and idiopathic pulmonary fibrosis (IPF) after an episode of pneumonia. The early identification of these patients would allow taking timely countermeasures. For this, specific markers (of clinical course, lung function, imaging, biomarkers, etc.) and therapeutic strategies need to be identified. Therefore, a deeper patho-mechanistic understanding of the pulmonary long-term sequelae of CAP is needed.

Extra-pulmonary long-term consequences of CAP (see also “Long-term cardio-vascular complications”): Severe pulmonary and systemic inflammation upon lung infection may result in long-term sequelae regarding organ dysfunction, vascular pathology, and neuromuscular function. Some patho-mechanistic links have been proposed, e.g. direct cardiac damage by bacterial invasion into the myocardium and formation of microscopic lesions finally leading to cardiac scarring [150] and a causal relationship between pulmonary inflammation and atherosclerotic plaque formation in systemic arteries [151]. However, many key patho-mechanisms by which pneumonia may trigger or promote subsequent organ dysfunction remain unclear. The complexity of the interplay between pulmonary inflammation and distant organ pathology and the relatively long timeframes render preclinical as well as clinical investigations in this field challenging. Nevertheless, the emerging evidence for the relevance of long-term sequelae for patient outcome and the probability for potentially effective secondary prophylactic measures warrant intense joint scientific efforts.

#### Comorbidities

##### Current setting

Pre-existing comorbidities including chronic respiratory, cardiovascular diseases and diabetes mellitus are frequent in the elderly population and increase the risk of CAP as well as mortality [152, 153]. Immunosenescence and therapies with immunosuppressive agents increase the number of immunosuppressed patients. Immunosuppression has been recognized as an independent risk factor for CAP [9]. Although the prevalence of different comorbidities in CAP patients has been evaluated across several studies, data on their impact on the course of the disease as well as their management during CAP are limited. Furthermore, most of the international guidelines on CAP management clearly state that the proposed recommendations do not apply to patients with immunosuppression [5]. International and observational studies on immunocompromised patients are limited or consider only a single specific risk factor. A direct implication of this scenario is the possible underestimation of the real prevalence of immunosuppression with a higher rate of treatment failure or an overestimation and overuse of wide-spectrum antibiotics.

##### Unmet needs

Treatment guidelines for immunocompromised patients: Up to 29% of hospitalized patients with CAP have some level of immunosuppression [154] and it is foreseeable that this population will expand in the following years. These patients may be at risk of both the “core” CAP pathogens and opportunistic microorganisms. Unfortunately, such patients are often excluded from pneumonia studies resulting in a marked knowledge gap concerning causative pathogens, performance of existing prognostic risk scores and performance of advanced diagnostic such as metagenomics among others. Immunocompromised patients do not form a homogeneous group in terms of underlying disease, treatment and severity of immunosuppression and the different immunosuppression states in the context of CAP need to be defined. It is possible that severely immunocompromised patients with CAP may benefit from adjunctive therapies to enhance specific functions of the immune system. Therefore, future studies need to focus on patients with risk factors for immunodeficiency in order to provide clinicians with recommendations for the management of immunocompromised patients with CAP.

Treatment guidelines for patients on special medication (i.e. patients on chronic steroids, biological drugs and cancer patients): Patients suffering from pre-existing lung diseases such as asthma or COPD are often treated with either inhaled or systemic corticosteroids [155]. Although these drugs reduce inflammation and might prevent exacerbations, the impact of the routine use of corticosteroids on CAP patients has not been investigated to full extent [156]. Biological drugs targeting tumour immune evasive pathways for the treatment of lung cancer [157] may guide different treatment regimens of CAP due to a changed immune status in these patients. With the ever-increasing number of patients suffering from pre-existing lung disease, it will be necessary to develop adjusted guidelines and treatment recommendations.

Influence of CAP on dementia and neurological damage: As in sepsis, during the acute phase of pneumonia, confusion is frequently observed [158, 159]. Evidence suggests that delirium may hasten cognitive deterioration in people with pre-existing dementia. Since pneumonia is primarily a disease of older patients there is the possibility that an episode of pneumonia will cause neurological damage and that the early onset of worsening of pre-existing dementia is a long-term consequence of CAP that has been overlooked so far.

#### Long-term cardio-vascular complications

##### Current setting

Hospitalized CAP patients have an increased risk of major acute and long-term cardiovascular complications (i.e. new/worsening heart failure, new/worsening arrhythmias, myocardial infarctions and/or strokes) [151, 160], which are associated with a 60% increase in short-term mortality [161]. Mortality remains increased even in long-term survivors of pneumonia [27, 162], an effect that has at least in part been attributed to an increased risk for cardiovascular diseases such as heart failure or atherosclerosis [151, 163, 164]. As a result of both acute and long-term effects, the ensuing risk for cardiovascular events associated with pneumonia is similar to or higher as compared to classic cardiovascular risk factors such as smoking or diabetes [165]. The underlying pathophysiological processes, however, are so far largely unclear.

##### Unmet needs

Spectrum, incidence and outcome of cardio-vascular disease after pneumonia: While there is clearly emerging evidence from epidemiological and preclinical studies for the association of pneumonia with both short- and long-term cardiovascular events, a comprehensive analysis of the various clinical manifestations (e.g. systolic or diastolic heart failure, arrhythmias, atherosclerotic and ischemic events) of cardiovascular disease (CVD), their incidence at different time points following a pneumonia event, and their association with outcome is as yet lacking, but would be critical to develop better diagnostic tools and ultimately tailor interventional trials.

Patients at increased risk of developing cardio-vascular disease after pneumonia: Patients with pre-existing chronic CVD have the strongest risk, followed by patients with common comorbidities including chronic obstructive pulmonary disease (COPD), ischemic heart disease, and diabetes [160]. The influence of these risk factors prior to the event of pneumonia, however, has not been stratified. Similarly, genetic traits associated with an increased risk for CVD after pneumonia have so far not been assessed. Furthermore, most studies focus so far on short-term outcomes of CAP. Cardiovascular complications associated with CAP may occur up to 10 years or more after the event of pneumonia. It would be helpful to stratify known cardiovascular risk factors to identify patients at an increased risk of developing CVD as early as possible and to develop therapeutic interventions to reduce the incidence of cardiac complications following CAP. Similarly, it would be helpful to identify microbial characteristics, and/or markers of pneumonia severity and host response that may predict association with CVD and CVD-related mortality [166].

Prevention of cardio-vascular disease after pneumonia: At present, our understanding of the patho-mechanisms that drive acute and chronic CVD following pneumonia is at best rudimentary. Discussed mechanisms include but are not limited to invasion of microbes such as *S. pneumoniae* into the myocardium with formation of microscopic lesions [150], or dissemination of inflammatory cells or pro-inflammatory mediators and extracellular vesicles driving inflammatory cardiomyopathic syndromes or formation and destabilization of atherosclerotic plaques [151]. A detailed in-depth understanding of these mechanisms by comparative systems medicine approaches in patient cohorts and preclinical models is required to inform the development and testing of targeted interventions to prevent CVD in patients-at-risk and/or in specific responder subgroups and as such, to create personalized therapeutic approaches.

## Conclusion

Pneumonia has been known as the leading infectious cause of death for more than a century and many attempts have been made to change this fact. However, the mortality of CAP has barely changed in the last 50 years and the Covid-19 pandemic has once again highlighted the central importance of acute respiratory infections to human health. Pneumonia is not just an infection of the lung but a complex disease where the interplay between the pathogen(s), immune system and comorbidities not only impose an immediate risk of mortality but also affect the patients’ risk of developing comorbidities as well as mortality for up to a decade after the pneumonia has resolved. Despite the importance of pneumonia on human health and the fact that many of the identified topics have been focus points for several years, our review of unmet needs in CAP research has shown that there are still major shortcomings in our knowledge of CAP. The poor evidence base that exists for most clinical decisions in acute respiratory infections can no longer be considered acceptable and a co-ordinated focus and investment into research on acute respiratory infections is now needed.

## Take home message

Unmet needs have been identified for diagnostics, risk stratification, treatment, adjunctive therapy, and prevention. Major knowledge gaps include immune response, role of comorbidities, and long-term cardio-vascular complications.

## Data Availability

Not applicable.

## Abbreviations

CVD: Cardiovascular disease
CAP: Community-acquired pneumonia
CT: Computer tomography
CAC: Coronary artery calcium
COPD: Chronic obstructive pulmonary disease
COP: Cryptogenic organizing pneumonia
HAP: Hospital-acquired pneumonia
IPF: Idiopathic pulmonary fibrosis
ICU: Intensive care unit
JAK: Janus kinase
MDR: Multidrug resistant
CAPNETZ: Network of excellence on Community Acquired Pneumonia
PCVs: Pneumococcal conjugate vaccines
PPVs: Pneumococcal polysaccharide vaccines
qSOFA: Quick SOFA
RSV: Respiratory syncytial virus
VAP: Ventilator-associated pneumonia
PCV7: 7-Valent conjugate vaccine
PCV13: 13-Valent conjugate vaccine
